# L'ostéotomie de scarf dans le traitement de l'hallux valgus: à propos de 19 cas

**DOI:** 10.11604/pamj.2014.19.189.4317

**Published:** 2014-10-23

**Authors:** Hassan Boussakri, Mohammed Bachiri, Mohammed Elidrissi, Mohammed Shimi, Abdelhalim Elibrahimi, Abdelmajid Elmrini

**Affiliations:** 1 Service de Chirurgie Ostéoarticulaire B4, CHU Hassan II, Université Sidi Mohammed Ben Abdellah, 3000 Fez, Maroc

**Keywords:** Hallux valgus, ostéotomie, Scarf, Hallux valgus, osteotomy, Scarf

## Abstract

L'ostéotomie de Scarf constitue une technique chirurgicale bien décrite, grâce à ses résultats excellents, elle est considérée comme un traitement de choix de l'hallux valgus. Le but de ce travail est de décrire le profil épidémiologique et radio-clinique des hallux valgus, ainsi qu’évaluer les résultats radiologiques et fonctionnels des patients traités par la technique classique d'ostéotomie de Scarf. Nous avons mené une étude rétrospective, concernant 22 ostéotomies de Scarf chez 19 patients, opérés d'hallux valgus entre mai 2009 et janvier 2013. Le recul moyen était de 22,5 mois (3-42 mois). L'âge moyen des patients au moment de l'intervention était de 49 ans, avec des extrêmes de 19 et 75 ans. Tous les patients ont bénéficié d'une évaluation clinique et radiologique préopératoire et postopératoire ainsi qu'une analyse statistique. Au dernier recul, selon des critères subjectifs, nos résultats étaient très satisfaisants dans 42%, satisfaisants dans 52% et déçus dans 6%, et en fonction du score de l'AOFAS, les résultats étaient nettement améliorée avec un AOFAS préopératoire de 57% à 84% en postopératoire. Concernant les résultats radiologiques, la déformation métatarsophalangienne (angle M1P1) a été corrigée (43,63 °-12,8°) avec une p très significative (p <0,001). D'autre part une amélioration de l'Angle moyen M1M2, passer de 18,18° préopératoire à 12,95° au dernier recul, avec une correction significative (p <0,001). Le valgus épiphysaire de premier métatarsien (AADM) a été statistiquement amélioré (p <0,001), passer de 24,45° à 7,91°. Concernant les complications nous avons noté un cas de sepsis précoce superficiel géré par une antibiothérapie adaptée, deux cas de névrome et un cas d'ostéonécrose. Par contre on n'a pas noté aucune fracture per opératoire du premier métatarsien. Nous concluons que l'ostéotomie de Scarf est une technique reproductible fiable, en pleine évolution.

## Introduction

L′hallux valgus est une affection fréquente [1], elle touche le plus souvent la Femme que l'homme. Le traitement chirurgical fait l'objet de nombreuses communications et discussions scientifiques, avec plusieurs procédures thérapeutiques décrites [2]. Mais l'ostéotomie de Scarf reste la technique chirurgicale de choix, même recommandée dans les hallux valgus modéré à sévère [3]. Le but de ce travail est de décrire le profil épidémiologique et radio-clinique des hallux valgus ainsi qu’évaluer les résultats radiologiques et fonctionnels des patients traités par la technique classique d'ostéotomie de Scarf [4].

## Méthodes

Entre mai 2009 et janvier 2013, une étude rétrospective a été menée, concernant 22 ostéotomies de scarf chez 19 patients, colligé au service de chirurgie osteoarticulaire B4 de FES –MAROC-. L’âge moyen de nos patients est 49 ans, avec des extrêmes de 19 et 75 ans, un écart type de 15 et une médiane de 50. à noter que 47,36% des patients étaient de plus de 50 ans. Une nette prédominance féminine a été notée dans 74% des cas, et un pied égyptien dans 81%. Le recul moyen est de 22,5 mois, avec des extrêmes de 3-42 mois et un écart type de 16 et une médiane de 27. Nous avons inclus dans cette étude tous les malades présentants un hallux valgus douloureux traité par une ostéotomie de scarf, et nous avons exclus tous les hallux valgus traités par autre technique que le Scarf.

**Figure 1:**
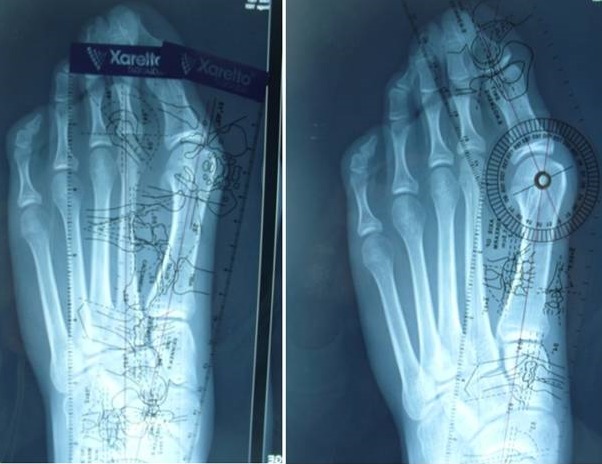
L’étude radiologique sur des radiographies standards à l'aide un goniomètre

Nous avons effectué une évaluation clinique et radiologique préopératoire et au dernier recul. L’évaluation fonctionnelle basée sur des critères subjectifs notamment: la douleur, esthétique et à l'aide de score de AOFAS préopératoire et au dernier recul. L’étude radiologique faite sur des radiographies standards de face, qui nous a permis de mesurer les angles: métatarsophalangien (M1P1); métatarsus valgus(M1M2); valgus épiphysaire de premier métatarsien (AADM) a l'aide d'un goniomètre (Figure 1), cependant L'analyse statistique des résultats a été faite à l'aide de logiciel SPSS (version 2012).

### Technique opératoire

**Figure 2:**
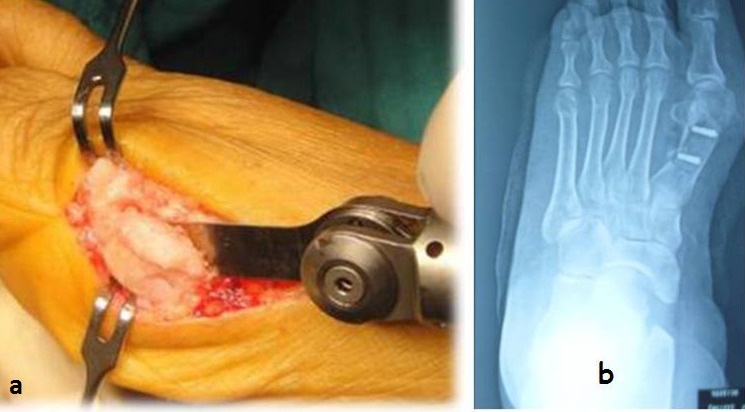
a) montrant voie d'abord chirurgicale ainsi que réalisation d'ostéotomie de Scarf; b) radiographie du pied montrant la fixation par deux vis de Scarf après ostéotomie de Scarf

Tous les patients ont été opérés par le même procédé opératoire, par un seul chirurgien senior, en décubitus dorsal et sous anesthésie locorégionale avec une antibioprophylaxie d'induction. Un garrot pneumatique à la racine de la cuisse était systématique. Nous avons utilisé des étapes classique d'ostéotomie de Scarf, par une voie d'abord interne, jonction peau plantaire et dorsale (Figure 2, A) puis La face interne de la première métatarsienne a été exposée, une double ostéotomie par une micro-scie oscillant a été réalisé (Figure 2, A), par la suite une translation et une fixation par des vis de Scarf (Figure 2, B). Par la même voie d'abord on réalise une section du tendon d'abducteur. À la fin de l'acte opératoire une capsuloplastie interne a été faite avec fermeture cutané.

Concernant les suites postopératoires, un simple pansement est réalisé avec une attelle postérieure. La prophylaxie Thromboembolique n′a pas été systématiquement administrée sauf chez les patients à haut risque Thromboembolique. Les patients de notre série ont été autorisés à marcher partiellement sur les talons immédiatement, à 2 semaines ablation de plâtre et mise en place des chaussures de Barouk avec appui complet en charge autorisé à 8 semaines.

## Résultats

**Figure 3:**
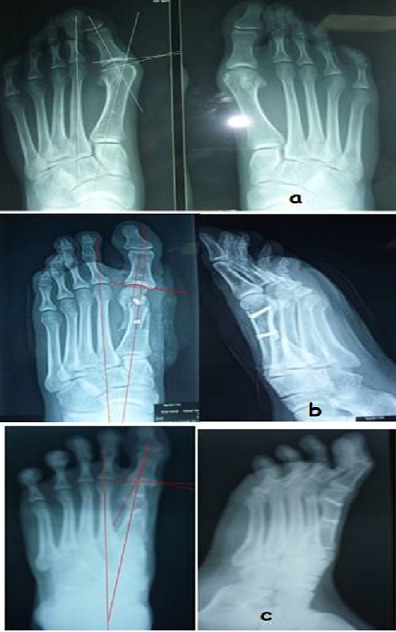
Résultats anatomiques de l'observation n°11 (B.0); a) aspect radiologique préopératoire; b) radiographie standard postopératoire immédiate; c) contrôle radiologique au recul d'un an

**Figure 4:**
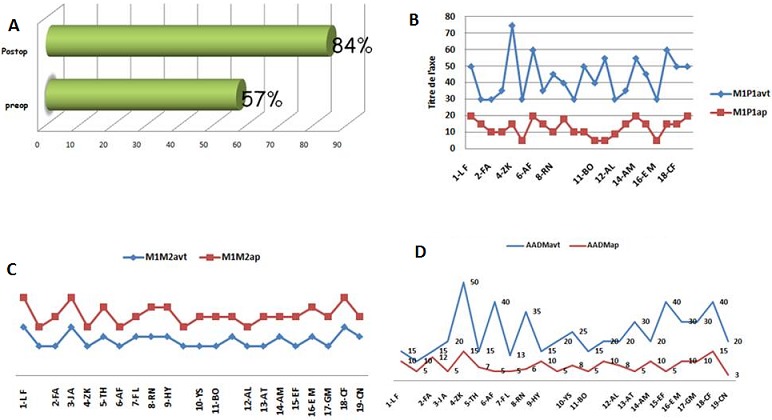
A) les résultats fonctionnels selon le score AOFAS préopératoire et postopératoire; B) mensuration de l'angle M1P1 préopératoire et postopératoire des patients de la série; C) mensuration de l'angle M1M2 préopératoire et postopératoire des patients de la série; D) comparaison de l'angle AADM préopératoire et postopératoire des patients de la série

Tous les patients ont bénéficié seulement d'une ostéotomie de Scarf classique sans autre geste associé type ostéotomie de la phalange proximal. Au dernier recul, Selon des critères subjectifs nos résultats étaient très satisfaisants dans 42%, satisfaisants dans 52% et déçus dans 6% (2 patients) (Figure 3). Par ailleurs selon le score de l'AOFAS, les résultats ont été nettement améliorés avec un score AOFAS préopératoire de 57 % à 84% en postopératoire (Figure 4, A). Concernant les résultats radiologique (Tableau 1), la déformation métatarsophalangienne a été corrigée avec un angle M1P1 moyen préopératoire de 43,63° passer à 12,8° en postopératoire, une moyenne 30,818, écart type de 11,245 avec une p très significative (p <0,001) (Figure 4, B et Tableau 2, Tableau 3). D'autre part une amélioration de l'Angle moyen M1M2, passer de 18,18° préopératoire a 12,95° au dernier recul, avec une Moyenne 5,227, écart type de 3,927avec une correction significative (Figure 4, C) et Tableau 4, Tableau 5). Le valgus épiphysaire de premier métatarsien AADM été de 24,45° passer au 7,91° au dernier recul avec une Moyenne 16,545, écart type de 10,299 et L'amélioration était statistiquement significative (p <0,001) (Figure 4, D) et Tableau 6, Tableau 7).

**Tableau 1:** Montrant les résultats anatomiques par des mensurations des différents angles préopératoire et au dernier recul

Patients	M1P1 préopératoire	M1M2 préopératoire	AADM préopératoire	M1P1 révision	M1M2 révision	AADM révision
1-L F	50	25	15	20	15	10
	30	15	10	15	10	5
2-FA	30	15	15	10	15	12
3-J A	35	25	20	10	15	5
4-ZK	75	15	50	15	10	15
5-TH	30	20	15	5	15	7
6-AF	60	15	40	20	10	5
7-F L	35	20	13	15	10	5
8-RN	45	20	35	10	15	6
9-HY	40	20	15	18	15	10
	30	15	20	10	10	5
10-YS	50	15	25	10	15	8
11-BO	40	15	15	5	15	5
	55	20	20	5	10	10
12-AL	30	15	20	9	10	8
13-AT	35	15	30	15	15	5
14-AM	55	20	20	20	10	10
15-EF	45	15	40	15	15	5
16-E M	30	20	30	5	15	10
17-GM	60	15	30	15	15	10
18-CF	50	25	40	15	15	15
19-CN	**50**	20	20	20	10	3

**Tableau 2:** Analyse statistique par logiciel SPSS de l'angle M1P1 préopératoire et postopératoire

Statistiques pour échantillons appariés		Moyenne	N	Ecart-type	Erreur standard moyenne
Paire 1	M1P1avt	**43,64**	22	12,553	2,676
	M1P1ap	**12,82**	22	5,188	1,106

**Tableau 3:** Analyse statistique par logiciel SPSS de l'angle M1P1 préopératoire et postopératoire (1)

Test échantillons appariés		Différences appariées					t	ddl	Sig. (bilatérale)
		Moyenne	Ecart-type	Erreur standard moyenne	Intervalle de confiance 95% de la différence				
					Inférieure	Supérieure			
Paire 1	M1P1avt - M1P1ap	30,818	11,245	2,397	25,833	35,804	12,855	21	0,000

**Tableau 4:** Analyse statistique par logiciel SPSS de l'angle M1M2 préopératoire et postopératoire

Statistiques pour échantillons appariés		Moyenne	N	Ecart-type	Erreur standard moyenne
Paire 1	M1M2avt	18,18	22	3,634	0,775
	M1M2ap	12,95	22	2,516	0,536

**Tableau 5:** Analyse statistique par logiciel SPSS de l'angle M1M2 préopératoire et postopératoire

Test échantillons appariés		Différences appariées					t	ddl	Sig. (bilatérale)
		Moyenne	Ecart-type	Erreur standard moyenne	Intervalle de confiance 95% de la différence				
					Inférieure	Supérieure			
Paire 1	M1M2avt - M1M2ap	5,227	3,927	0,837	3,486	6,968	6,243	21	0,000

**Tableau 6:** Analyse statistique par logiciel SPSS de l'angle AADM préopératoire et postopératoire

Statistiques pour échantillons appariés		Moyenne	N	Ecart-type	Erreur standard moyenne
Paire 1	AADMavt	24,45	22	10,883	2,320
	AADMap	7,91	22	3,379	0,720

**Tableau 7:** Analyse statistique par logiciel SPSS de l'angle AADM préopératoire et postopératoire (1)

Test échantillons appariés		Différences appariées					t	ddl	Sig. (bilatérale)
		Moyenne	Ecart-type	Erreur standard moyenne	Intervalle de confiance 95% de la différence				
					Inférieure	Supérieure			
Paire 1	AADMavt - AADMap	16,545	10,299	2,196	11,979	21,112	7,535	21	0,000

**Figure 5:**
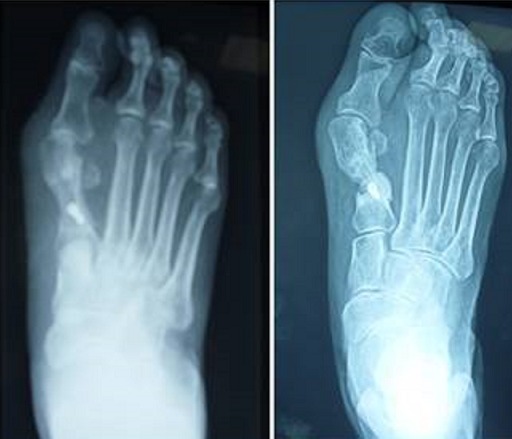
Le seul cas d'ostéonécrose: à gauche radiographie postopératoire après 2 mois et à droite radiographie après 8 mois de recul

Concernant les complications nous avons noté un cas de sepsis précoce superficiel géré par une antibiothérapie adaptée à l'antibiogramme associée à des changements de pansements quotidiens, deux cas de névrome et un cas d'ostéonécrose (Figure 5), par contre on n'a pas noté aucune fracture peropératoire du premier métatarsien.

## Discussion

L′hallux valgus est une déformation complexe, progressive dans le temps, affectant l′avant-pied, se manifeste cliniquement par une déviation latérale de grande orteil. L'ostéotomie de Scarf est une technique fiable et permet une correction significative des déformations des hallux valgus modérée à sévère [3, 5].

Meyer [6] est parmi les premiers qui ont décrit les grands principes de cette technique chirurgicale, Mais l'arrivée de Weil et Borelli [7] aux Etats-Unis et Barouk [8, 9] en France ont contribués au développement de cette chirurgie. Cependant plusieurs Modifications de l′ostéotomie de Scarf ont été décrit [4, 10], mais ont tous en commun une ostéotomie de la première métatarsienne type Z avec une fixation interne et une capsulorraphie médiane [11]. L'ostéosynthèse permet une compression rigide de la zone d'ostéotomie aboutissant à une consolidation osseuse et permettant un retour rapide à la mise en charge et une activité normale pour éviter un déplacement secondaire voir une pseudarthrose ainsi qu'une diminution des amplitudes articulaire grâce a une stabilité satisfaisante [12, 13]. Les objectifs du traitement chirurgicale sont la correction de la déformation et l'obtenir des articulations indolores, libres et mobiles, sans oublier d'obtenir un bon aspect esthétique (Figure 2, B). Nos résultats fonctionnels et cliniques sont comparables et identiques à ceux de la littérature [14, 15], à noter que la majorité des publications de la littérature soulignent La faible corrélation entre le score AOFAS global et les résultats radiologiques, d′autres insistent que le score AOFAS n'est pas suffisant pour l’évaluation des résultats, d'où la nécessité d'autres scores complémentaires d’évaluation [1, 14, 16].

Concernant nos résultats radiologiques, il y ‘avait une amélioration moyenne de l'angle M1P1 de 12,8° (à partir de 43,63 °avec extrême de 75° à 30°) et de M1M2 de 12,95° (à partir de 18,18° avec extrême de 25°à 15°). Ces résultats sont comparables à ceux publiés dans la littérature [1, 16, 17]. D'autre part Le valgus épiphysaire du premier métatarsien AADM est passé de 24,45° à 7,91° au dernier recul avec extrêmes de 5°à 15°, Cependant il existe une controverse en ce qui concerne la mesure de la correction de l'angle AADM. Certains auteurs rapportent que La correction du AADM n′était pas significative après ostéotomie de Scarf et ont expliqué ce constat par, le plus important c'est la restauration de l′os et l′alignement des articulations que réalignement de la surface cartilagineuse du métatarsien, en plus la difficulté d’évaluer l'angle AADM sur des radiographie standard [1]. Alors que Sullivan et al [18] ont montré que peu de corrélation entre la mensuration préopératoire et postopératoire de l'angle AADM sur une radiographie standard après ostéotomie de Scarf [1, 16, 19]. Par contre Notre étude ainsi que D'autre études de la littérature [14], notamment La série de Ajay et all [3], ont montré une correction significative de l'angle AADM, tout en insistant sur le fait que l’ostéotomie de Scarf permet une correction de rotation.

Les complications rapportées concernant cette technique chirurgicale comprennent l′infection, des métatarsalgies, une ostéonécrose de la tête de première métatarsienne, une fracture du premier métatarsien et une algodystrophie [20]. Dans notre série, globalement le taux de complications est faible et rejoint celui de la littérature [9, 20].

## Conclusion

Certes, notre étude présente de nombreuses limites, notamment c'est une étude rétrospective, d'autre part c'est une série limitée dans le temps et dans le nombre, cependant Nous concluons que l'ostéotomie de Scarf est une technique reproductible fiable, en pleine évolution: l'ostéotomie Scarf-évolution.
